# A Novel Ion Channel Formed by Interaction of TRPML3 with TRPV5

**DOI:** 10.1371/journal.pone.0058174

**Published:** 2013-02-28

**Authors:** Zhaohua Guo, Christian Grimm, Lars Becker, Anthony J. Ricci, Stefan Heller

**Affiliations:** 1 Departments of Otolaryngology – HNS and Molecular & Cellular Physiology, Stanford University School of Medicine, Palo Alto, California, United States of America; 2 Department of Pharmacy – Center for Drug Research and Center for Integrated Protein Science Munich, Ludwig-Maximilians-Universität, München, Germany; Sackler Medical School, Tel Aviv University, Israel

## Abstract

TRPML3 and TRPV5 are members of the mucolipin (TRPML) and TRPV subfamilies of transient receptor potential (TRP) cation channels. Based on sequence similarities of the pore forming regions and on structure-function evidence, we hypothesized that the pore forming domains of TRPML and TRPV5/TRPV6 channels have similarities that indicate possible functional interactions between these TRP channel subfamilies. Here we show that TRPML3 and TRPV5 associate to form a novel heteromeric ion channel. This novel conductance is detectable under conditions that do not activate either TRPML3 or TRPV5. It has pharmacological similarity with TRPML3 and requires functional TRPML3 as well as functional TRPV5. Single channel analyses revealed that TRPML3 and TRPV5 heteromers have different features than the respective homomers, and furthermore, that they occur in potentially distinct stoichiometric configurations. Based on overlapping expression of TRPML3 and TRPV5 in the kidney and the inner ear, we propose that TRPML3 and TRPV5 heteromers could have a biological function in these organs.

## Introduction

The TRP cation channel TRPML3 is expressed by dermal melanocytes, by vomeronasal and olfactory neurons, and in the inner ear by marginal cells of the stria vascularis as well as sensory hair cells [1], [2]. Based on RT-PCR and expressed sequence tag analyses, TRPML3 mRNA is also detectable in various other organs, most prominently in the kidney, thymus, and various glands [3]. TRPML3 belongs to the mucolipin subfamily of TRP channels, which also includes TRPML1 and TRPML2. Subcellularly, all TRPML channels are located mainly in lysosomal and endosomal vesicles, although this tendency appears to be most prominent for TRPML1 and TRPML2, which harbor lysosomal targeting sequences. TRPML3 is able to associate with TRPML1, which affects intracellular trafficking of the presumptively heteromeric channels to intracellular compartments [4]. Mutations in the human *TRPML1* gene cause Mucolipidosis Type IV, a lysosomal storage disorder [5], [6]. A point mutation in the murine *Trpml3* gene causes the varitint-waddler (*Va*) phenotype manifested in pigmentation defects, circling behavior, and hearing loss [7]. The *Va* mutation causes a constitutively open channel, resulting in elevated [Ca^2+^]_i_, which ultimately leads to apoptotic death of cells expressing TRPML3 [1], [8]–[10]. Interestingly, two independently generated targeted knockout mouse lines of the *Trpml3* gene exhibit neither inner ear dysfunction nor pigmentation defects, nor any other obvious phenotypes [2], [11]. It has been hypothesized that one of the possibilities for the lack of a phenotype in *Trpml3* mutant mice could be that TRPML3 might serve as subunit of unknown heteromeric channels [11]. The observed heteromerization of TRPML3 with its related TRP subfamily members TRPML1 and TRPML2 [12]–[14] supports this hypothesis.

Here, we show that TRPML channels are able to closely associate with TRPV5 and TRPV6, two distinct members of the TRPV subfamily of TRP channels whose known roles are in Ca^2+^ reabsorption in the kidney and intestine. TRPML channels are also expressed in the kidney and intestine [3], whereas TRPV5 and TRPV6 have also been reported in the inner ear; particularly TRPV5 has been detected in marginal cells of the stria vascularis and in cochlear sensory hair cells [15], although the expression in hair cells was not observed in another study [16]. Based on this overlapping expression, particularly in the kidney and the inner ear, we focused our attention on investigating whether the association of TRPML3 with TRPV5 leads to a novel conductance. We used whole cell recordings, pharmacology, co-expression with mutant channel isoforms, and single channel analyses to provide evidence for the existence of a heteromeric novel ion channel formed by TRPML3 and TRPV5 subunits.

## Results

### TRPML and TRPV5/6 Channels Closely Associate

**Figure 1:**
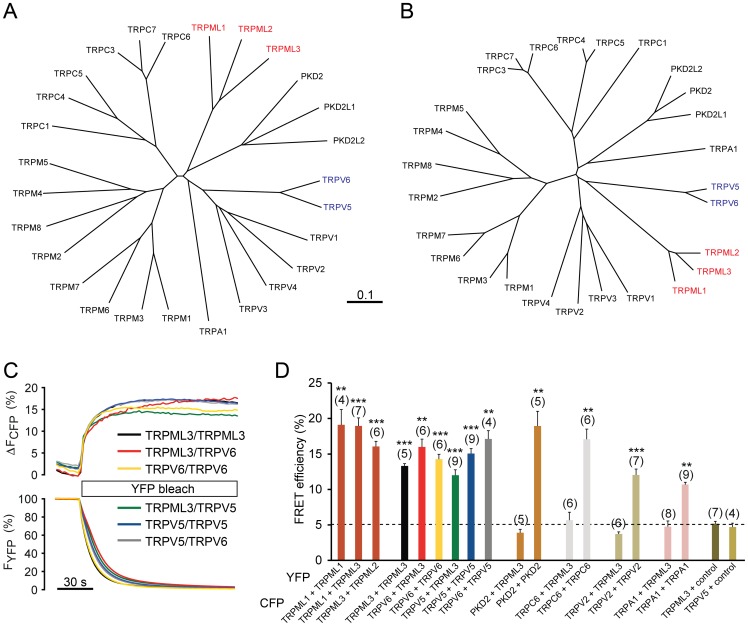
Interaction between TRPML channels and TRPV5/6. (A) Phylogenetic tree, based on full length sequence comparisons of human TRP channel proteins. (B) Phylogenetic tree, based on sequence comparisons of the pore forming domains of human TRP channel proteins. (C) Fluorescence energy resonance transfer (FRET) experiments showing representative FRET efficiencies among TRPML3, TRPV5, and TRPV6 channels. FRET efficiencies were determined by measuring the recovery of CFP fluorescence during YFP photobleaching. (D) Average FRET efficiencies reported as mean values± SEM (n = parenthesized). Shown are efficiencies for TRPML, TRPV5, and TRPV6 channel homo- and heteromers, as well as PKD2, TRPC6, TRPV2, and TRPA1 as controls. YFP and CFP indicate the fluorescent tag, which was carboxyl-terminal in all cases. Control indicates a pcDNA3.1-based expression vector for the corresponding fluorescent protein (YFP in the example shown). ***p<0.0001 and **p<0.001, Student’s t-test, unpaired, comparison with TRPC6/TRPML3 coexpression as negative control.

Functional analyses of the *Va* mutant isoform of TRPML3 revealed susceptibility for helix-breaking mutations in the fifth transmembrane-spanning domain (TM5), which results in constitutive channel activity [1], [8]–[10]. The propensity for pore property alteration by helix-breaking mutations is a common feature of TRPML channels and surprisingly also for the two TRPV family members TRPV5 and TRPV6 [8], [17]. Based on these previous findings, we became intrigued by a potential similitude of TRPML with TRPV5 and TRPV6 channels, particularly with respect to the pore region. At first glance, based on overall protein sequence homology, TRPML and TRPV channels are quite dissimilar and form discrete subfamilies (Fig. 1A). When we conducted a sequence comparison that was restricted to the pore regions including sequences from the start of TM5 to the end of TM6, however, we found that TRPV5 and TRPV6 formed a distinct subfamily that was phylogenetically closer to the TRPML channel subfamily than to the TRPV1-4 subfamily cluster (Fig. 1B). A closer look at the individual sequences revealed that the similarity of TRPML channels with TRPV5 and TRPV6 is greatest for the stretch of amino acids comprising TM5 (Fig. S1). This sequence similarity combined with the previous observations of susceptibility for helix-breaking mutations raised our curiosity whether TRPML and TRPV5/6 channels might be able to interact physically. We investigated whether co-expressed TRPML and TRPV5/6 channels are in close enough association to display Förster/fluorescence resonance energy transfer (FRET). Homomeric pairs of cyan fluorescent protein (CFP) and yellow fluorescent protein (YFP)-tagged TRPML channels as well as TRPV5 and TRPV6 showed FRET efficiencies between 14–22% (Fig. 1C,D), indicative of close proximity of the two fluorophores. Previously known heteromeric channel pairs such as TRPML1/TRPML3, TRPML2/TRPML3, and TRPV5/TRPV6, as well as homomeric TRPML1, TRPML3, TRPV5, TRPV6, PKD2, TRPC6, TRPV2, and TRPA1 channels displayed equally pronounced FRET efficiencies ranging from 10–20%. All combinations of TRPML1 and TRPML3 with TRPV5 and TRPV6 also exhibited FRET ranging from 12–17% efficiency. Negative controls including pairs of TRPML3 with PKD2, TRPC6, TRPV2, and TRPA1 or YFP alone showed no FRET, indicated by thresholds below 5%. These results suggested that TRPML3 is able to closely associate with TRPV5 and TRPV6.

### TRPML3 forms a novel conductance when coexpressed with TRPV5

**Figure 2:**
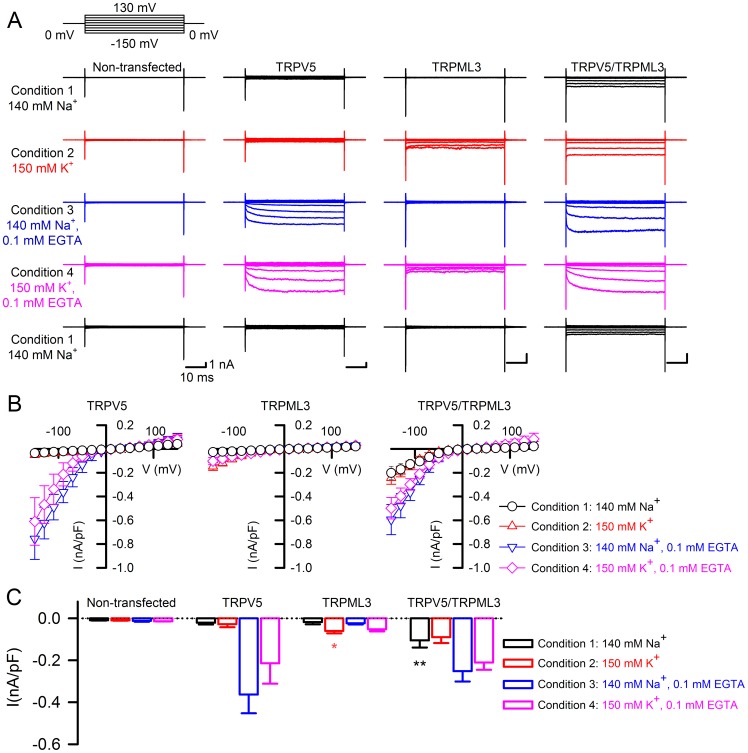
Whole-cell currents of cells expressing TRPV5, TRPML3, and both proteins in different ionic conditions. (A) Traces show representative currents obtained from non-transfected HEK293 cells, and cells expressing TRPV5, TRPML3, and TRPV5/TRPML3, in the presence of extracellular solutions starting with 140 mM Na^+^ (condition 1, black), and successively switched to 150 mM K^+^ (condition 2, red), 140 mM Na^+^, 0.1 mM EGTA (condition 3, blue), followed by 150 mM K^+^, 0.1 mM EGTA (condition 4, pink), and a final set of measurements conducted in condition 1. Currents were recorded during voltage steps from −150 mV to +130 mV in 20 mV increments, holding at 0 mV. (B) Mean values (±SD) of average inward current densities of TRPV5 (n = 10), TRPML3 (n = 12), and TRPV5/TRPML3 (n = 10) plotted against voltage in the presence of 140 Na^+^ (black circles), 150 K^+^ (red triangles), 140 Na^+^, 0.1 EGTA (blue triangles), and 150 K^+^, 0.1 EGTA (magenta diamonds), respectively. (C) Average inward current densities at −90 mV of HEK293 cells expressing the different channels in the four different conditions as indicated. Bar diagrams represent mean ± SD, numbers in parentheses are the number of cells analyzed. ** p<0.001 and * p<0.01, Student’s t-test, unpaired.

We next examined whether the suggested association of TRPML3 and TRPV5/6 channels becomes functionally manifest in whole cell patch clamp experiments using transfected HEK293 cells. For these analyses, we decided to focus on TRPML3 and TRPV5 and to investigate channel properties under four different conditions, which were selected to distinguish between TRPML3 and TRPV5 activities: under condition 1, both channels were expected to be inactive, condition 2 enables TRPML3 currents, condition 3 leads to activation of TRPV5, and condition 4 was picked for activation of both channels. Condition 1 is standard bath solution containing 140 mM Na^+^ as the major cation, 5 mM K^+^, and 1.5 mM Ca^2+^. Under this condition, TRPV5 and TRPML3 are inactive, and as expected, we did not record any considerable currents when either channel was expressed alone (Fig. 2A,B). In cells cotransfected with TRPV5 and TRPML3 expression plasmids, however, we were able to elicit inwardly rectified currents when we stepped to negative voltages. At −150 mV, in standard bath solution, we measured a current density of −0.129±0.052 nA/pF (mean±SD, n = 10) for this novel current. Condition 2 contained K^+^ as the major cation (150 mM K^+^), a low concentration of Na^+^ (2 mM), and 1.5 mM Ca^2+^. As expected, TRPV5 was inactive in this condition and TRPML3 displayed inwardly rectifying currents as shown previously [3], [18] with a current density of −0.10±0.006 nA/pF (n = 12) at −150 mV. Cotransfected cells exhibited larger currents than TRPML3-only expressing cells with an average current density of −0.169±0.067 nA/pF (n = 10) at −150 mV. Under condition 3, containing 140 mM Na^+^, 5 mM K^+^, and 0.1 mM EGTA, which favors active TRPV5 and inactive TRPML3, we measured TRPV5 activity at −150 mV of −0.54±0.12 nA/pF (n = 10); TRPML3 was inactive, as expected. Cotransfected cells displayed average currents of −0.326±0.007 nA/pF (n = 10) at −150 mV. Finally, under condition 4, containing 150 mM K^+^, 2 mM Na^+^, and 0.1 mM EGTA, which enables TRPV5 and TRPML3 to be active, we measured average current densities at −150 mV for TRPV5 of −0.379±0.1 nA/pF, for TRPML3 of 0.07±0.02 nA/pF, and for the cotransfected cells: −0.283±0.09 nA/pF (n = 10, 12, and 10, respectively). Our whole cell recording experiments show that HEK293 cells cotransfected with expression plasmids for TRPV5 and TRPML3 display a novel conductance that is active under conditions where the homomeric channels are inactive.

### The novel conductance is pharmacologically closer to TRPML3 and requires stoichiometric contributions of TRPV5 and TRPML3

**Figure 3:**
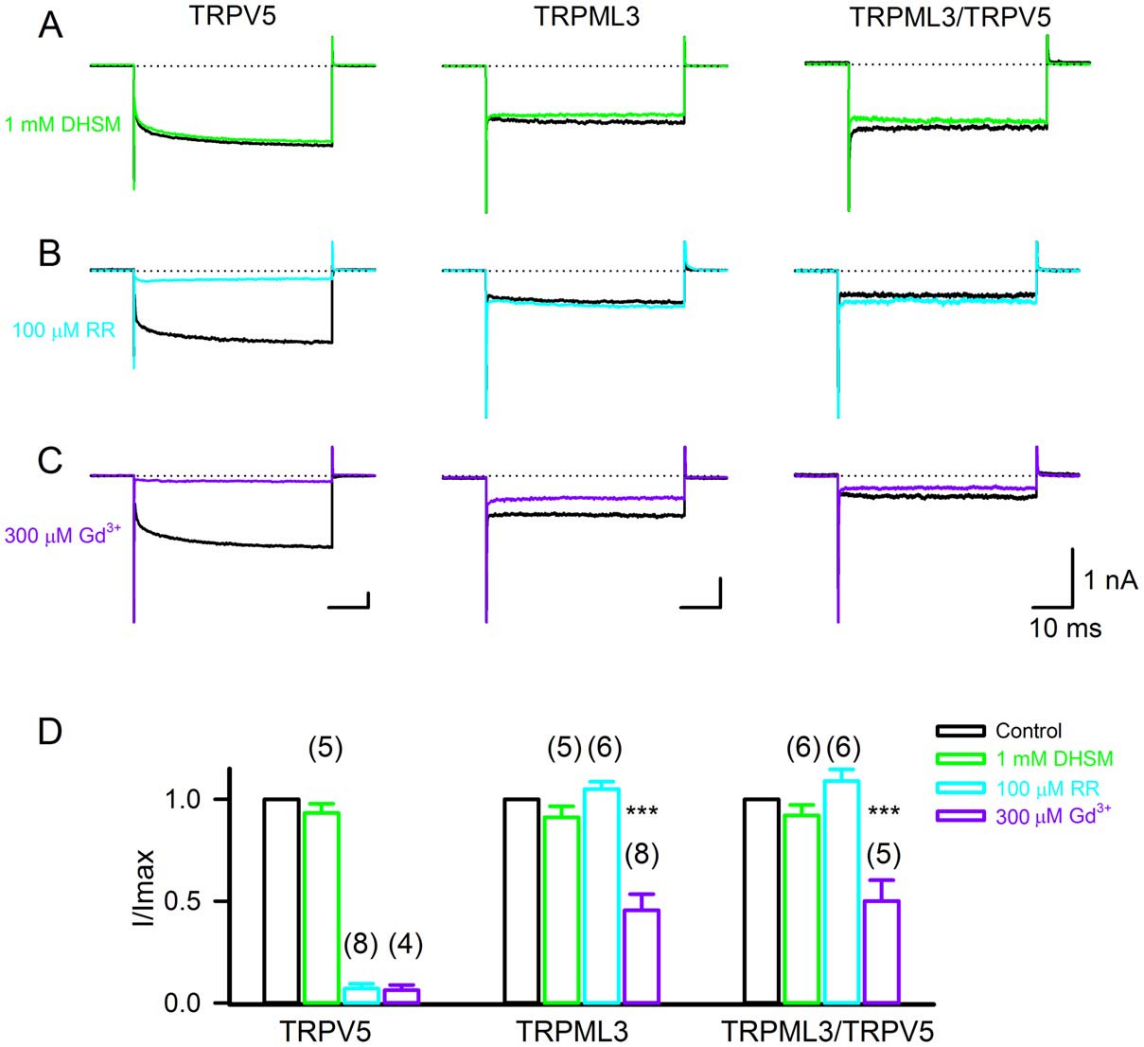
Pharmacological properties of HEK293 cells expressing TRPV5, TRPML3, and both proteins. (A–C) Representative traces shown from transfected HEK293 cells expressing TRPV5, TRPML3, and TRPV5/TRPML3 in response to step polarization (from 0 mV to –150 mV) before (black lines) and after 1 mM dihydrostreptomycin (DHSM) (green lines), 100 µM Ruthenium Red (RR) (cyan lines) and 300 µM gadolinium chloride (Gd^3+^) (purple lines). (D) Quantitative analysis of the percentage of inhibition at −150 mV (mean±SD, n = 4–8).

To characterize the novel conductance pharmacologically, various blockers were used. We employed the inner ear sensory hair cell mechanoelectrical transduction channel blocker dihydrostreptomycin (DHSM, [19], [20]), because TRPV and TRPML channels have been discussed as potential hair cell transduction channel candidates [21], [22]. DHSM, at 1 mM, did not block TRPV5, TRPML3, or the novel conductance (Fig. 3A,D). Ruthenium red is a potent blocker of TRPV5 [23], [24] and at 100 µM, the reagent abolished more than 90% of the TRPV5 current elicited at −150 mV (Fig. 3B,D). TRPML3-based currents were not affected by ruthenium red, nor was the novel conductance. Gadolinium ion, a non-specific cation channel blocker that affects many TRP channels as well as mechanosensitive channels [25], blocked more than 90% of TRPV5 currents and partially blocked TRPML3 (Fig. 3C,D, see also [1]). The novel conductance was also partially blocked by gadolinium ion. Based on these pharmacological assessments, we conclude that the novel conductance has no pharmacological resemblance with TRPV5, but for the blockers tested, it resembles TRPML3. Nevertheless, this result has to be interpreted with caution because no specific blockers, particularly for TRPML3 are available.

**Figure 4:**
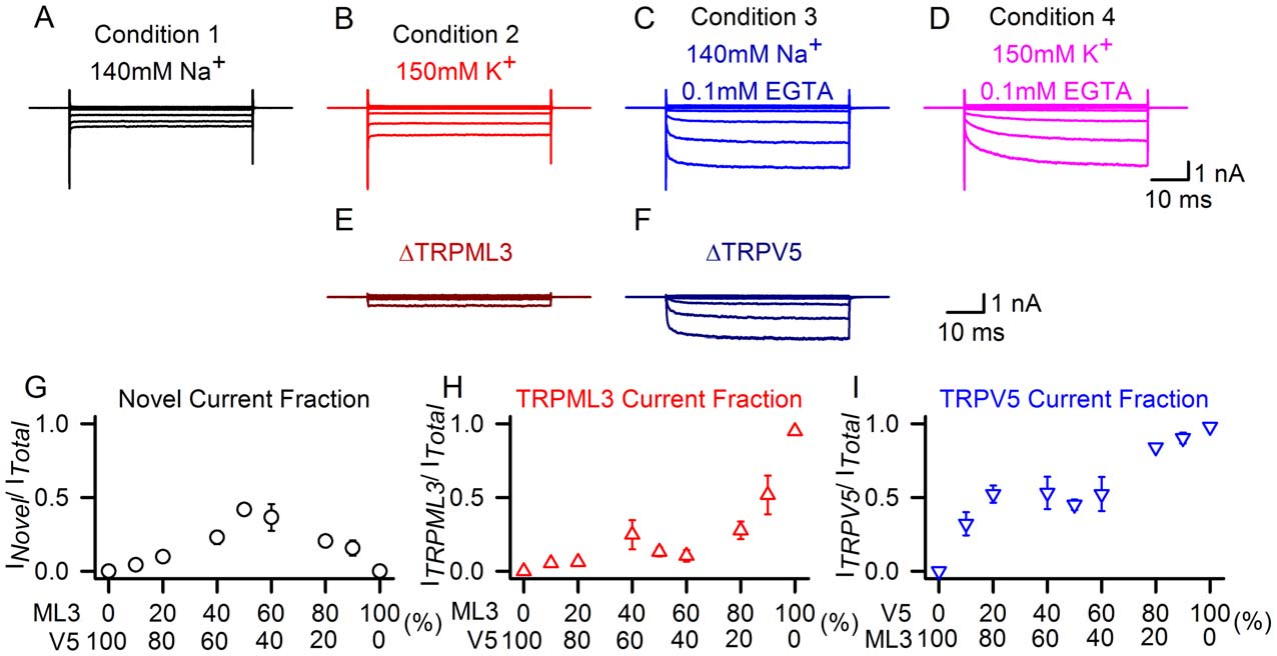
Stoichiometric analyses of TRPML3/TRPV5 currents. (A–D) Traces show currents at −150 mV recorded from HEK293 cells expressing TRPV5/TRPML3, in the presence of extracellular solutions containing 140 mM Na^+^ (condition 1, black), 150 mM K^+^ (condition 2, red), 140 mM Na^+^, 0.1 mM EGTA (condition 3, blue), and 150 mM K^+^, 0.1 mM EGTA (condition 4, pink). We hypothesized that the current recorded under condition 1 represents the novel conductance, the current under condition 2 consists of the novel conductance and TRPML3, the current under condition 3 is composed of the novel conductance and TRPV5, and finally, under condition 4, we expected that the recorded current consists of all three conductances. (E–F) Subtraction of A from B (in E) which represents pure TRPML3; subtraction of A from C (in F) which results in pure TRPV5. (G–I) HEK293 cells were transfected with TRPML3 and TRPV5 expression plasmids either pure (0% and 100%) and at molar ratios 1∶10 (10%), 1∶5 (20%), 2∶3 (40%), 1∶1 (50%), 3∶2 (60%), 5∶1 (80%) and 10∶1 (90%), respectively. Currents were elicited at −150 mV under conditions 1–3 and individual conductances for TRPML3 and TRPV5 were extracted as described in (E–F). Shown is the average fraction of novel current (G, black circles), TRPML3 (H, red triangles), and TRPV5 (I, blue triangles) is plotted against the fraction of TRPML3 over TRPV5 (G,H) and TRPV5 over TRPML3 (I) (n = 5–11).

To gain more insight into the interaction between TRPV5 and TRPML3, we transfected HEK293 cells with different ratios of plasmid DNA encoding the individual TRP proteins. We measured whole cell currents of the cotransfected cell populations under the four different conditions described above (Fig. 4A–D), and determined the contribution of individual conductances by subtracting the currents measured in the different conditions. For example, the contribution of TRPML3-based currents under condition 2 (Fig. 4B, at 150 mM K^+^) can be determined by subtracting the measured currents under condition 1 (Fig. 4A, at 140 mM Na^+^), resulting in TRPML3 (Fig. 4E). Subtracting condition 1 (Fig. 4A) from condition 3 (Fig. 4C), results in TRPV5 (Fig. 4F). Plotting the individual components for each current at different TRPV5:TRPML3 ratios revealed that the majority of the novel conductance was detected when the two channels were expressed at approximately equal levels (Fig. 4G). Shifting the ratio towards TRPML3 resulted in major contribution of TRPML3-based current of the total current (Fig. 4H), and expectedly, at a ratio that favors TRPV5, the main current of the total was TRPV5-based (Fig. 4I). Overall, if one of the two TRP channels was present at 80% or higher, very little of the new conductance was measured. These results suggest that the novel conductance requires a roughly equal contribution of TRPML3 and TRPV5.

### Coexpression with inactive and dominant-negative isoforms suggests that the novel conductance is a heteromeric ion channel

**Figure 5:**
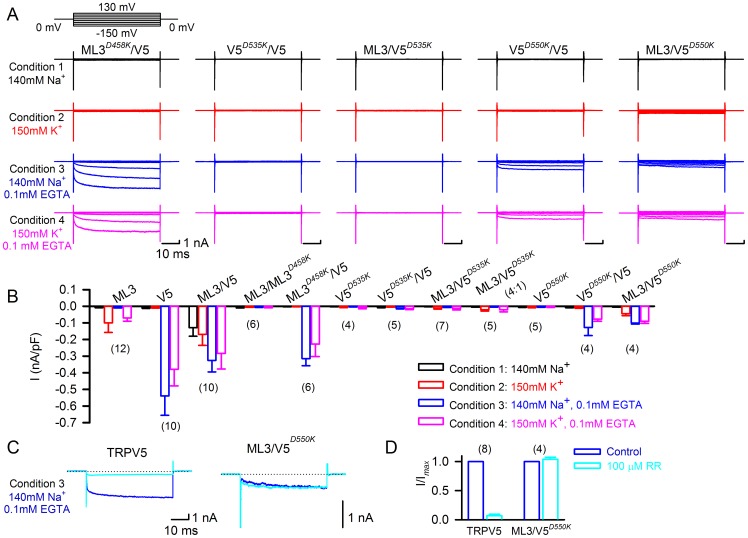
Effects of dominant-negative and inactive isoforms of TRPML3 and TRPV5. (A) Representative whole cell currents shown were obtained from cells coexpressing TRPML3^D458K^ and TRPV5 (ML3^D458K^/ V5), TRPV5^D535K^ and TRPV5 (V5^D535K^/ V5), TRPML3 and TRPV5^D535K^ (ML3/V5^D535K^), TRPV5^D550K^ and TRPV5 (V5^D550K^/ V5), and TRPML3 and TRPV5^D550K^ (ML3/V5^D550K^) under the four conditions indicated. Currents were recorded during voltage steps from −150 mV to +130 mV in 20 mV increments, holding at 0 mV. (B) Average inward current densities at −150 mV of the various TRPML3 and TRPV5 channel pairs under the four different conditions as indicated. Bar diagrams represent mean±SD, numbers in parentheses are the number of cells analyzed. (**C**) Pharmacological properties of HEK293 cells expressing TRPV5 and TRPML3/TRPV5^D550K^ (ML3/V5^D550K^). Representative traces were recorded during voltage steps from 0 mV to −150 mV under condition 3 before (blue) and after (cyan) application of 100 µM ruthenium red (RR). (**D**) Quantitative analysis of the percentage of inhibition at −150 mV (mean±SD, n = parenthesized).

Our results thus far are compatible with two scenarios. Either, TRPV5 and TRPML3 form a heteromeric channel in which both TRP proteins contribute to the channel pore or, the novel conductance is TRPML3-based and simply modulated (activated) by close association with TRPV5. To gain more insight into these two possible scenarios, we investigated several mutant isoforms of TRPML3 and TRPV5 and coexpressed them with the wild type channels in equal ratios. TRPML3(D458K) is a dominant-negative isoform of TRPML3 [3], [4], and when co-expressed with TRPML3, it abolishes all TRPML3-based currents (Fig. 5B: ML3/ML3^D458K^). When coexpressed with TRPV5, we neither recorded channel activity in TRPML3-enabling condition 2 (as expected), nor did we detect the novel current under condition 1 (Fig. 5A,B: ML3^D458K^/V5). TRPV5-based currents were still measurable under conditions 3 and 4. These results suggest that functional TRPML3 is needed for the novel conductance. It also shows that dominant-negative TRPML3, when expressed in equal ratio with TRPV5 is not able to abolish presumptive TRPV5 homomeric channel activity. Overall, this result is compatible with both scenarios.

TRPV5^D535K^ is an inactive TRPV5 isoform [26] that, when coexpressed with TRPV5, suppresses activity (Fig. 5A,B: V5^D535K^/V5). Based on this observation, we conclude that TRPV5^D535K^ is dominant-negative. When coexpressed with TRPML3 (ML3/V5^D535K^), no channel activity was detectable under any of the conditions investigated. This result suggests that the novel conductance (condition 1) requires functional TRPV5. The fact that no wild type TRPML3 current was detectable under condition 2 suggests a direct dominant-negative interaction of TRPV5^D535K^ with TRPML3, which is highly indicative of heteromerization. Changing the ratio of TRPML3 to TRPV5^D535K^ to 80% over 20%, allowed us to titer the dominant-negative effect to the point that homomeric TRPML3 became detectable (ML3/V5^D535K^ (4∶1)), suggesting that TRPV5^D535K^ has similar ability to associate with TRPML3 as wild type TRPV5 (Fig. 4H–J). Overall, these results imply that the novel conductance is the product of a TRPV5:TRPML3 heteromer.

We also investigated TRPV5^D550K^, an inactive but not dominant-negative isoform (Fig. 5A,B: V5^D550K^ and V5^D550K^/V5). Coexpressed with TRPML3, this isoform did not lead to detectable currents under condition 1 (Fig. 5A,B: ML3/V5^D550K^), confirming that the novel conductance requires functional TRPV5. As expected, we recorded a current in TRPML3-enabling condition 2. Surprisingly, in TRPV5-enabling conditions 3 and 4, we also measured a current. This current was not blockable with 100 µM ruthenium red (Fig. 5C), indicating that it is different from TRPV5 homomeric current. These results show that the inactive TRPV5^D550K^ isoform is able to functionally interact with wild type TRPML3, leading to a channel that is active in TRPV5-enabling, but normally not TRPML3-enabling condition 3. Albeit suggestive that these observations point towards a potential heteromeric interaction, they are also compatible with a possible modulatory role of TRPV5 on TRPML3.

### Single channel analyses of the novel conductance

**Figure 6:**
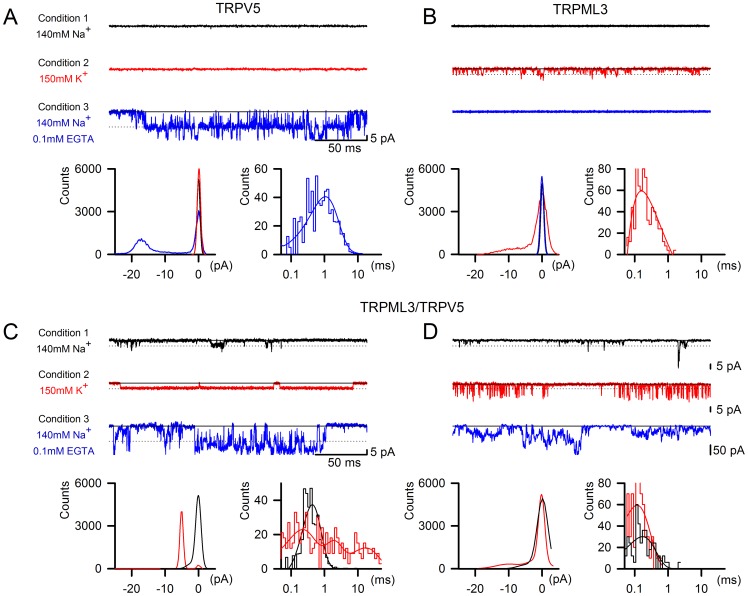
Single channel recordings of TRPV5, TRPML3, and TRPML3/TRPV5 currents. Shown are representative single-channel traces, as well as amplitude and open dwell-time histograms for TRPV5 (A), TRPML3 (B), and the two varieties of traces observed for TRPML3/TRPV5 (C,D) under conditions 1, 2, and 3 at −150 mV. Solid lines and dotted lines represent closed and open averaged, respectively. The open dwell-time histograms were fitted to biexponential functions.

Single channel analyses in outside-out patches from plasmid-transfected HEK293 cells revealed obvious differences among TRPV5, TRPML3, and the novel conductance. TRPV5 was not active under conditions 1 and 2, but under condition 3 at −150 mV, it displayed relatively large single channel events with a conductance of 105.27±5.4 pS (n = 6) and average open times of 1.02±0.12 ms (Fig. 6A). This single channel behavior is in agreement with previous reports [27], [28]. TRPML3 also showed expected behavior and was only active under conditions 2 and 4, with an average conductance under condition 2 of 32.7±1.0 pS at −150 mV, and an average open time of 0.22±0.06 ms (n = 5) (Fig. 6B).

Because neither TRPV5 nor TRPML3 were active under condition 1, we hypothesized that single channel events observed with cotransfected cells under this condition were attributable to the novel conductance. Two kinds of distinctively different single channel events were detectable at equal ratios for TRPML3 and TRPV5 cotransfected cells (Fig. 6C,D). The first current, which we observed in 4 of 8 cases had an average conductance of 32.3±0.8 pS at −150 mV in condition 1 (Fig. 6C). The average open time was 0.40±0.02 ms. Notably, changing the outside patch solution to condition 2 resulted in substantially prolonged open times. Whereas the average conductance did not change substantially (33.4±0.4 pS), the mean open time was significantly increased to 5.98±1.37 ms (p<0.001). Addition of 0.1 mM EGTA, which generates TRPV5-enabling conditions 3 and 4, further affected the novel conductance’s single channel features by becoming more flickery. The increased flickering, the larger amplitudes, and the occurrence of multiple events under conditions 3 and 4 suggested a presence of TRPV5 homomers that were silent under conditions 1 and 2, which made it impossible to deduce exact current amplitudes. The second type of current, which we observed in 4 of 8 cases is shown in Fig. 6D, and displayed average single channel conductances of 34.6±1.2 pS and 46.7±3.4 pS with average open times of 0.16±0.05 ms and 0.17±0.02 ms in conditions 1 and 2, respectively.

Overall, the single channel analyses revealed that two types of novel conductances form when TRPML3 and TRPV5 are co-expressed. Both types of conductances are distinctively different from the individual homomeric channels either by conductance or by average open time.

## Discussion

TRP channels consist of four subunits that are assembled into a common tetrameric structure featuring a central pore. Heteromerization of TRP channels is not very common in vertebrates and if it happens, it has mainly been observed among closely related members of individual subfamilies, for example between members of the TRPC subfamily [29], [30], among TRPML channels [13], [31], or between TRPV5 and TRPV6 [32]. Heteromeric interactions between TRP channels of different subfamilies are even less common, but have been reported for TRPP2 and TRPC1 [33], [34] as well as for TRPP2 and TRPV4 [35], [36]. Another example is the observation that TRPV1 is able to closely associate with TRPA1 and that TRPV1 modulates the single channel characteristics of TRPA1, albeit without heteromerization [37].

It is apparent that heteromerization would substantially increase diversity of TRP channels. If all 28 mammalian TRP channels were able to pairwise heteromultimerize, more than 268 million different combinations would be possible. It is obvious that such a scenario is not the case and that vertebrate TRP channels have evolved mostly as homomeric channels with specific, albeit sometimes quite multifunctional properties. The infrequent occurrence raises the question whether heteromerization is of biological importance (see [30] for a discussion of this topic). For TRPML channels, heteromerization affects several physiological processes such as intracellular and plasma membrane trafficking [3], [31], autophagy [13], as well as gene expression [38]. TRPP2 and TRPV4 are both essential components of the ciliary flow sensor in renal epithelial cells, and the channels jointly mediate thermosensation in double knockout mice [36]. These examples suggest that heteromeric TRP channels do play important physiological roles, which justifies further investigation of their properties.

Our rationale for investigating a potential heteromerization of TRPML with TRPV5 and TRPV6 channels started with the observation that specific pore-altering mutations in transmembrane-spanning domain 5, which lead to constitutive activation of TRPML channels had similar effects on TRPV5 and TRPV6, but not on a variety of other TRP channels [8]. A possible explanation for this similarity is that the pore forming regions of the two channel groups have structural resemblance, which is supported by the sequence comparisons shown in Fig. 1. Our FRET experiments showed that TRPML3 is able to closely associate with other TRPML channels and with TRPV5 and TRPV6, but not with other TRP channels including TRPP2, a channel that has been involved in the majority of previously reported cross-subfamily heteromeric TRP channel assemblies [33]–[36]. Coexpression of TRPML3 with TRPV5 revealed a novel conductance in HEK293 cells under conditions in which the respective homomeric channels are inactive; a similar conductance was also observed when we co-expressed TRPML3 with TRPV6 (Fig. S2). Recorded whole cell currents were inwardly rectifying and exhibited a cation channel. The novel conductance was not blocked by ruthenium red, but its current was significantly reduced when exposed to gadolinium ion, suggesting a potential resemblance with TRPML3’s features. Neither TRPML3 and TRPV5 homomers nor the heteromeric current were inhibited by the sensory hair cell mechanoelectrical transduction channel blocker dihydrostreptomycin, indicating that none of the conductances are likely to play a role in hair cell mechanotransduction, clarifying previous speculations [21], [22]. Our stoichiometric analysis suggests that the novel conductance is only detectable when TRPML3 and TRPV5 are expressed more or less equally; excess of a single subunit leads to disappearance of the novel current, which requires functional TRPML3 as well as functional TRPV5. These analyses are compatible with two possible scenarios. The first scenario is that coexpression of TRPV5 modulates the activity of TRPML3 resulting in a TRPML3-like current that is no longer blocked by sodium and therefore is active under condition 1 featuring 140 mM Na^+^. The second scenario is that the novel conductance is a true heteromer consisting of TRPML3 and TRPV5 subunits. Compatible with both scenarios is the finding that inactive TRPV5^D550K^ when co-expressed with TRPML3 results in a current under conditions that specifically enable TRPV5 activity. This activity is not blocked with ruthenium red, which indicates presence of a channel with novel features or a modulated TRPML3 conductance. Evidence for heteromerization, however, is the fact that a dominant-negative TRPV5 subunit is able to inhibit TRPML3. Dominant-negative effects, however, can also be exerted when a mutant isoform diverts trafficking of the heteromer to intracellular compartments.

Additional support for heteromerization of TRPML3 with TRPV5 comes from our single channel analyses, where we found two types of channel activity that were distinctively different from TRPML3 and TRPV5. Under condition 1 (140 mM Na^+^) in which neither TRPML3 nor TRPV5 are normally active, TRPML3/TRPV5 transfected cells had membrane patches that harbored single channels with conductances between 32–35 pS, similar to the single channel conductance measured for TRPML3, and a little less than 1/3 of the single channel conductance of TRPV5. Average open times of these novel conductances were 0.16 ms and 0.4 ms, delineating two groups of single channels, one of which with significantly longer open times than homomeric TRPML3 (0.22 ms, when measured under condition 2).

Switching to condition 2 (150 mM K^+^) revealed that half of the single channels that were detectable under condition 1 displayed significantly longer open times that increased from an average of 0.4 ms to 5.98 ms, but maintained the single channel conductance of 33 pS. The other half of single channels, when switched from condition 1 to condition 2, did not change open times, but we noticed a greater tendency to flicker and an increase of average conductance of the single channels from 35 pS to 47 pS. The observed change in opening time of the first group of channels is reminiscent of the ability of Na^+^ to block TRPML3 [3], [18], [39], and could be attributable to the TRPML3 component of the heteromeric channel. The fact that two distinct novel channels were detectable in equal distributions indicates that putative heteromers exist in different configurations. Albeit speculative, one could imagine tetramers with single TRPML3 or TRPV5 subunits, or tetramers with equal numbers of TRPML3 and TRPV5 subunits. The investigation of such different stoichiometric configurations requires more refined additional analyses that could utilize specific features of the individual subunits, for example the relatively large first extracellular loop of TRPML3, which might be distinguishable in atomic force microscopy as recently utilized in characterization of TRPP2 and TRPV4 heteromers [35].

Heteromerization of TRPML3 with TRPV5, as demonstrated in our study, is rather unexpected because the individual homomeric channels appear unrelated at first glance. TRPV5 is Ca^2+^-selective, which enables its physiological function of Ca^2+^-reabsorption in the kidney [40]. TRPML3 on the other hand, albeit inwardly rectifying like TRPV5, is a nonselective cation channel [10], which has been proposed to shuttle between the plasma membrane and intracellular compartments such as endosomes and lysosomes [3], [31], [41]. Expression of both channels overlaps in renal epithelial cells as well as in the cochlea, where both channels have been reported in sensory hair cells and in cells of the stria vascularis [2], [3], [15], [42]. Based on the premise that the two channel proteins are co-expressed in cells of the kidney and the inner ear, it is possible that TRPML3 and TRPV5 heteromers play biological roles in these organs. Future work needs to focus on elucidating whether TRPML3 and TRPV5 heteromers are present and physiologically active in native cells in these organs, research that is hampered by the lack of tools such as antibodies that work for immunoprecipitation of native proteins.

## Materials and Methods

### Sequence comparisons

Phylogenetic trees of the human TRP channel proteins based on amino acid alignments using either full-length sequences or the sequences comprising TM5, pore domain, and TM6 were generated with DNAMAN software (Lynnon Corporation, Pointe-Claire, Quebec, Canada) in combination with NJplot (http://pbil.univ-lyon1.fr/software/njplot.html). The accession numbers and the sequences for the human TRP proteins used in the analysis are listed in Table S1. Alternatively, sequences were analyzed with the Clustal V and Clustal W methods using the MegAlign tool of the DNASTAR software (DNASTAR, Madison, WI, USA), which produced comparable results.

### Förster/fluorescence resonance energy transfer

Fluorescence resonance energy transfer (FRET) measurements were performed with the iMic platform and Polychrome V monochromator (TILL Photonics, Martinsried, Germany). Human embryonic kidney cells (HEK293, ATCC No. CRC-1573) were transiently transfected with the respective YFP and CFP fusion constructs. FRET efficiencies were determined by measuring the recovery of CFP fluorescence during YFP photobleaching. Cells were excited at 410 nm and 515 nm for CFP and YFP detection, respectively. YFP was bleached with 512 nm light.

### Heterologous expression

HEK293 cells were grown in 75 cm^2^ cell culture flasks in 90% (v/v) Minimum Essential Medium (Gibco), 10% (v/v) heat inactivated fetal bovine serum, and 100 µg/ml penicillin, and routinely passaged when 80% confluent. 24 h before transfection, the cells were passaged in 6-well cell culture plates onto glass coverslips (No.1 22×30 mM, Warner Instruments), which were precut to about 7×7 mm, sterilized, and coated with poly-L-lysine (50 µg/ml). Transfection was conducted using 3 µl GeneJammer transfection reagent (Stratagene) and 5 µl plasmid DNA at a concentration of 200 ng/µl in a total volume of 97 µl MEM. After 18–20 hr, the cells were supplied with fresh growth media and used for electrophysiological analyses. Expression plasmids were based on pcDNA3.1 (Invitrogen), containing full-length mouse TRPML3 (GenBank accession number NM_134160), human TRPML3 (NM_018298) and full-length mouse TRPV5 (NM_001007572) sequence, carboxyl-terminally fused to either yellow fluorescent protein (YFP)- or cyan fluorescent protein (CFP). YFP- or CFP- negative cells were used as non-transfected controls. Site-directed mutagenesis was performed on wild-type cDNA templates using the QuikChange Site-Directed Mutagenesis Kit (Stratagene). All mutants were verified by sequencing both strands entirely.

### Electrophysiology

Currents were recorded in whole-cell configuration and for single channel analysis from outside-out patches. Pipettes of 2–4MΩ resistance were filled with an internal solution consisting of 140 mM CsCl, 10 mM Hepes, 4 mM ATP-Na, 10 mM BAPTA, and 1 mM MgCl2, adjusted to pH 7.2 with CsOH. All experiments were conducted at 22–25 C.

Giga seals were formed under condition 1 extracellular solution, which contained 140 mM NaCl, 5 mM KCl, 1 mM MgCl_2_, 1.5 mM CaCl_2_, 10 mM Hepes, and 10 mM D-glucose, adjusted to pH 7.4 with NaOH. Condition 2 extracellular solution consisted of 150 mM KCl, 2 mM NaCl, 1 mM MgCl_2_, 1.5 mM CaCl_2_, 10 mM Hepes, and 10 mM D-glucose, adjusted to pH 7.4 with KOH. Condition 3 comprised a nominally Ca^2+^-free, Mg^2+^ – free extracellular solution of 140 mM NaCl, 5 mM KCl, 0.1 mM EGTA, 10 mM Hepes, and 10 mM D-glucose, adjusted to pH 7.4 with NaOH. Condition 4 extracellular solution contained 150 mM KCl, 2 mM NaCl, 0.1 mM EGTA, 10 mM Hepes, and 10 mM D-glucose, adjusted to pH 7.4 with KOH. Chemicals were obtained from Thermo Fisher Scientific (Waltham, MA), Sigma-Aldrich Corporation (St. Louis, MO), and USB Products (Cleveland, OH).

Transfected cultured HEK293 cells were identified by fluorescence microscopy for plasma membrane targeted YFP-tagged TRPML3 and CFP-tagged TRPV5. Only cells of healthy appearance expressing the tagged channels at moderate levels, judged by fluorescence intensity, were used for patch clamp experiments. Recordings were conducted with an Axoclamp 200B amplifier (Axon Instruments, Foster City, CA; sampling rate 0.1 ms, 8-pole Bessel filter at 2 kHz) coupled to a Digidata-1440 board (Molecular Devices, Sunnyvale, CA) interfacing with a personal computer (Dell) running Windows XP (Microsoft Corporation, Seattle, WA). jClamp Software (Scisoft, New Haven, CT) was used for all data collection.

Solutions were applied locally onto the both whole-cell and outside-out patches via a puffer pipette with tip diameter of 100 µM at a distance of 100–120 µM. The flow rate was controlled by a Minipuls 3 peristaltic pump (Gilson Inc., Middleton, WI, USA) set at 1.5 rpm coupled through miniature solenoid valves (The Lee Company, Westbrook, CT), resulting in continuous perfusion with complete exchange of the bath solution in approximately 90 sec.

### Data analysis and statistics

Whole cell current analysis was done with Clampfit (Axon Instruments, Foster City, CA). Single channel currents and amplitudes were analyzed with the QUB software package (State University of New York, Buffalo, NY), which was also used for generation of histograms. Idealization was done using the segmentation k-means algorithm after digital low-pass filtering to 10 kHz. Kinetic modeling of the idealized intervals was done using the maximum interval likelihood method. Data presented are mean values± standard deviation (SD) with the number of independent experiments (n) indicated. Statistical differences were determined with unpaired two-tailed t tests using Sigma Plot 11.0 (SPSS Science, Chicago, IL) on a Dell personal computer running Windows XP.

## Supporting Information

Figure S1 **Alignments of TRP channel sequences spanning TM5, the pore loop, and TM6.** (**A**) Identical amino acids are shown with black background. (**B**) TM5-pore-TM6 sequences of human and mouse TRPV5 and TRPML3. The corresponding sequence of KcsA is shown for comparison.
(TIF)

Figure S2 **Whole-cell currents of cells expressing TRPV6, and TRPML3 and TRPV6 in different ionic conditions.** Traces show representative currents obtained from transfected HEK293 cells expressing TRPV6, and TRPML3 and TRPV6 in the presence of extracellular solutions containing 140 mM Na^+^ (condition 1, black), 150 mM K^+^ (condition 2, red), 140 mM Na^+^, 0.1 mM EGTA (condition 3, blue), and 150 mM K^+^, 0.1 mM EGTA (condition 4, pink). Currents were recorded during voltage steps from −150 mV to +130 mV in 20 mV increments, holding at 0mV.
(TIF)

Table S1 **Accession numbers and sequences of the human TRP protein TM5-pore domain-TM6 segments used in the multiple sequence alignments.**
(PDF)

## References

[1] Nagata K, Zheng L, Madathany T, Castiglioni AJ, Bartles JR, et al (2008) The varitint-waddler (Va) deafness mutation in TRPML3 generates constitutive, inward rectifying currents and causes cell degeneration. Proc Natl Acad Sci U S A 105: 353–358. 18162548 10.1073/pnas.0707963105 PMC2224216

[2] Castiglioni AJ, Remis NN, Flores EN, Garcia-Anoveros J (2011) Expression and vesicular localization of mouse Trpml3 in stria vascularis, hair cells, and vomeronasal and olfactory receptor neurons. J Comp Neurol 519: 1095–1114. 21344404 10.1002/cne.22554 PMC4105223

[3] Grimm C, Jors S, Saldanha SA, Obukhov AG, Pan B, et al (2010) Small molecule activators of TRPML3. Chem Biol 17: 135–148. 20189104 10.1016/j.chembiol.2009.12.016 PMC2834294

[4] Kim HJ, Soyombo AA, Tjon-Kon-Sang S, So I, Muallem S (2009) The Ca(2+) channel TRPML3 regulates membrane trafficking and autophagy. Traffic 10: 1157–1167. 19522758 10.1111/j.1600-0854.2009.00924.x PMC2993507

[5] Bargal R, Avidan N, Ben-Asher E, Olender Z, Zeigler M, et al (2000) Identification of the gene causing mucolipidosis type IV. Nat Genet 26: 118–123. 10973263 10.1038/79095

[6] Zeevi DA, Frumkin A, Bach G (2007) TRPML and lysosomal function. Biochim Biophys Acta 1772: 851–858. 17306511 10.1016/j.bbadis.2007.01.004

[7] Di Palma F, Belyantseva IA, Kim HJ, Vogt TF, Kachar B, et al (2002) Mutations in Mcoln3 associated with deafness and pigmentation defects in varitint-waddler (Va) mice. Proc Natl Acad Sci U S A 99: 14994–14999. 12403827 10.1073/pnas.222425399 PMC137533

[8] Grimm C, Cuajungco MP, van Aken AF, Schnee M, Jors S, et al (2007) A helix-breaking mutation in TRPML3 leads to constitutive activity underlying deafness in the varitint-waddler mouse. Proc Natl Acad Sci U S A 104: 19583–19588. 18048323 10.1073/pnas.0709846104 PMC2148332

[9] Kim HJ, Li Q, Tjon-Kon-Sang S, So I, Kiselyov K, et al (2007) Gain-of-function mutation in TRPML3 causes the mouse Varitint-Waddler phenotype. J Biol Chem 282: 36138–36142. 17962195 10.1074/jbc.C700190200

[10] Xu H, Delling M, Li L, Dong X, Clapham DE (2007) Activating mutation in a mucolipin transient receptor potential channel leads to melanocyte loss in varitint-waddler mice. Proc Natl Acad Sci U S A 104: 18321–18326. 17989217 10.1073/pnas.0709096104 PMC2084341

[11] Jors S, Grimm C, Becker L, Heller S (2010) Genetic inactivation of Trpml3 does not lead to hearing and vestibular impairment in mice. PLoS ONE 5: e14317. 21179200 10.1371/journal.pone.0014317 PMC3001452

[12] Venkatachalam K, Hofmann T, Montell C (2006) Lysosomal Localization of TRPML3 Depends on TRPML2 and the Mucolipidosis-associated Protein TRPML1. J Biol Chem 281: 17517–17527. 16606612 10.1074/jbc.M600807200 PMC4196876

[13] Zeevi DA, Lev S, Frumkin A, Minke B, Bach G (2010) Heteromultimeric TRPML channel assemblies play a crucial role in the regulation of cell viability models and starvation-induced autophagy. J Cell Sci 123: 3112–3124. 20736310 10.1242/jcs.067330 PMC2931605

[14] Curcio-Morelli C, Zhang P, Venugopal B, Charles FA, Browning MF, et al (2010) Functional multimerization of mucolipin channel proteins. J Cell Physiol 222: 328–335. 19885840 10.1002/jcp.21956

[15] Takumida M, Ishibashi T, Hamamoto T, Hirakawa K, Anniko M (2009) Age-dependent changes in the expression of klotho protein, TRPV5 and TRPV6 in mouse inner ear. Acta Otolaryngol 129: 1340–1350. 19922080 10.3109/00016480902725254

[16] Yamauchi D, Nakaya K, Raveendran NN, Harbidge DG, Singh R, et al (2010) Expression of epithelial calcium transport system in rat cochlea and vestibular labyrinth. BMC Physiol 10: 1. 20113508 10.1186/1472-6793-10-1 PMC2825184

[17] Lee KP, Nair AV, Grimm C, van Zeeland F, Heller S, et al (2010) A helix-breaking mutation in the epithelial Ca(2+) channel TRPV5 leads to reduced Ca(2+)-dependent inactivation. Cell Calcium 48: 275–287. 21035851 10.1016/j.ceca.2010.09.007 PMC3780571

[18] Grimm C, Jors S, Guo Z, Obukhov AG, Heller S (2012) Constitutive Activity of TRPML2 and TRPML3 Channels versus Activation by Low Extracellular Sodium and Small Molecules. J Biol Chem 287: 22701–22708. 22753890 10.1074/jbc.M112.368876 PMC3391124

[19] Kroese AB, van den Bercken J (1980) Dual action of ototoxic antibiotics on sensory hair cells. Nature 283: 395–397. 6153235 10.1038/283395a0

[20] Kroese AB, Das A, Hudspeth AJ (1989) Blockage of the transduction channels of hair cells in the bullfrog's sacculus by aminoglycoside antibiotics. Hear Res 37: 203–217. 2468634 10.1016/0378-5955(89)90023-3

[21] Cuajungco MP, Grimm C, Heller S (2007) TRP channels as candidates for hearing and balance abnormalities in vertebrates. Biochim Biophys Acta 1772: 1022–1027. 17300924 10.1016/j.bbadis.2007.01.002 PMC1961624

[22] Corey DP (2006) What is the hair cell transduction channel? J Physiol 576: 23–28. 16901942 10.1113/jphysiol.2006.116582 PMC1995642

[23] Hoenderop JG, Vennekens R, Muller D, Prenen J, Droogmans G, et al (2001) Function and expression of the epithelial Ca(2+) channel family: comparison of mammalian ECaC1 and 2. J Physiol 537: 747–761. 11744752 10.1111/j.1469-7793.2001.00747.x PMC2278984

[24] Nilius B, Prenen J, Vennekens R, Hoenderop JG, Bindels RJ, et al (2001) Pharmacological modulation of monovalent cation currents through the epithelial Ca2+ channel ECaC1. Br J Pharmacol 134: 453–462. 11588099 10.1038/sj.bjp.0704272 PMC1572972

[25] Ermakov YA, Kamaraju K, Sengupta K, Sukharev S (2010) Gadolinium ions block mechanosensitive channels by altering the packing and lateral pressure of anionic lipids. Biophys J 98: 1018–1027. 20303859 10.1016/j.bpj.2009.11.044 PMC2849073

[26] Nilius B, Vennekens R, Prenen J, Hoenderop JG, Droogmans G, et al (2001) The single pore residue Asp542 determines Ca2+ permeation and Mg2+ block of the epithelial Ca2+ channel. J Biol Chem 276: 1020–1025. 11035011 10.1074/jbc.M006184200

[27] Nilius B, Vennekens R, Prenen J, Hoenderop JG, Bindels RJ, et al (2000) Whole-cell and single channel monovalent cation currents through the novel rabbit epithelial Ca2+ channel ECaC. J Physiol 527 Pt 2: 239–248. 10.1111/j.1469-7793.2000.00239.x PMC2270079 10970426

[28] Cha SK, Jabbar W, Xie J, Huang CL (2007) Regulation of TRPV5 single-channel activity by intracellular pH. J Membr Biol 220: 79–85. 18004496 10.1007/s00232-007-9076-2

[29] Hofmann T, Schaefer M, Schultz G, Gudermann T (2002) Subunit composition of mammalian transient receptor potential channels in living cells. Proc Natl Acad Sci U S A 99: 7461–7466. 12032305 10.1073/pnas.102596199 PMC124253

[30] Schaefer M (2005) Homo- and heteromeric assembly of TRP channel subunits. Pflugers Arch 451: 35–42. 15971080 10.1007/s00424-005-1467-6

[31] Zeevi DA, Frumkin A, Offen-Glasner V, Kogot-Levin A, Bach G (2009) A potentially dynamic lysosomal role for the endogenous TRPML proteins. J Pathol. 10.1002/path.2587 19557826

[32] Hellwig N, Albrecht N, Harteneck C, Schultz G, Schaefer M (2005) Homo- and heteromeric assembly of TRPV channel subunits. J Cell Sci 118: 917–928. 15713749 10.1242/jcs.01675

[33] Kobori T, Smith GD, Sandford R, Edwardson JM (2009) The transient receptor potential channels TRPP2 and TRPC1 form a heterotetramer with a 2:2 stoichiometry and an alternating subunit arrangement. J Biol Chem 284: 35507–35513. 19850920 10.1074/jbc.M109.060228 PMC2790980

[34] Tsiokas L, Arnould T, Zhu C, Kim E, Walz G, et al (1999) Specific association of the gene product of PKD2 with the TRPC1 channel. Proc Natl Acad Sci U S A 96: 3934–3939. 10097141 10.1073/pnas.96.7.3934 PMC22398

[35] Stewart AP, Smith GD, Sandford RN, Edwardson JM (2010) Atomic force microscopy reveals the alternating subunit arrangement of the TRPP2-TRPV4 heterotetramer. Biophys J 99: 790–797. 20682256 10.1016/j.bpj.2010.05.012 PMC2913176

[36] Kottgen M, Buchholz B, Garcia-Gonzalez MA, Kotsis F, Fu X, et al (2008) TRPP2 and TRPV4 form a polymodal sensory channel complex. J Cell Biol 182: 437–447. 18695040 10.1083/jcb.200805124 PMC2500130

[37] Staruschenko A, Jeske NA, Akopian AN (2010) Contribution of TRPV1-TRPA1 interaction to the single channel properties of the TRPA1 channel. J Biol Chem 285: 15167–15177. 20231274 10.1074/jbc.M110.106153 PMC2865321

[38] Samie MA, Grimm C, Evans JA, Curcio-Morelli C, Heller S, et al (2009) The tissue-specific expression of TRPML2 (MCOLN-2) gene is influenced by the presence of TRPML1. Pflugers Arch 459: 79–91. 19763610 10.1007/s00424-009-0716-5 PMC2913554

[39] Kim HJ, Li Q, Tjon-Kon-Sang S, So I, Kiselyov K, et al (2008) A novel mode of TRPML3 regulation by extracytosolic pH absent in the varitint-waddler phenotype. Embo J 27: 1197–1205. 18369318 10.1038/emboj.2008.56 PMC2367400

[40] Hoenderop JG, van Leeuwen JP, van der Eerden BC, Kersten FF, van der Kemp AW, et al (2003) Renal Ca2+ wasting, hyperabsorption, and reduced bone thickness in mice lacking TRPV5. J Clin Invest 112: 1906–1914. 14679186 10.1172/JCI19826 PMC297001

[41] Grimm C, Hassan S, Wahl-Schott C, Biel M (2012) Role of Trpml and Tpc Channels in Endo-Lysosomal Cation Homeostasis. J Pharmacol Exp Ther. 10.1124/jpet.112.192880 22518024

[42] Takumida M, Anniko M (2009) Expression of transient receptor potential channel mucolipin (TRPML) and polycystine (TRPP) in the mouse inner ear. Acta Otolaryngol: 1–8. 10.3109/00016480903013593 20095091

