# Supplementary figures and images for: A Novel Ion Channel Formed by Interaction of TRPML3 with TRPV5
Source: PLoS One. 2013 Feb 28;8(2):e58174. doi: 10.1371/journal.pone.0058174 (PMC3585263; doi:10.1371/journal.pone.0058174)

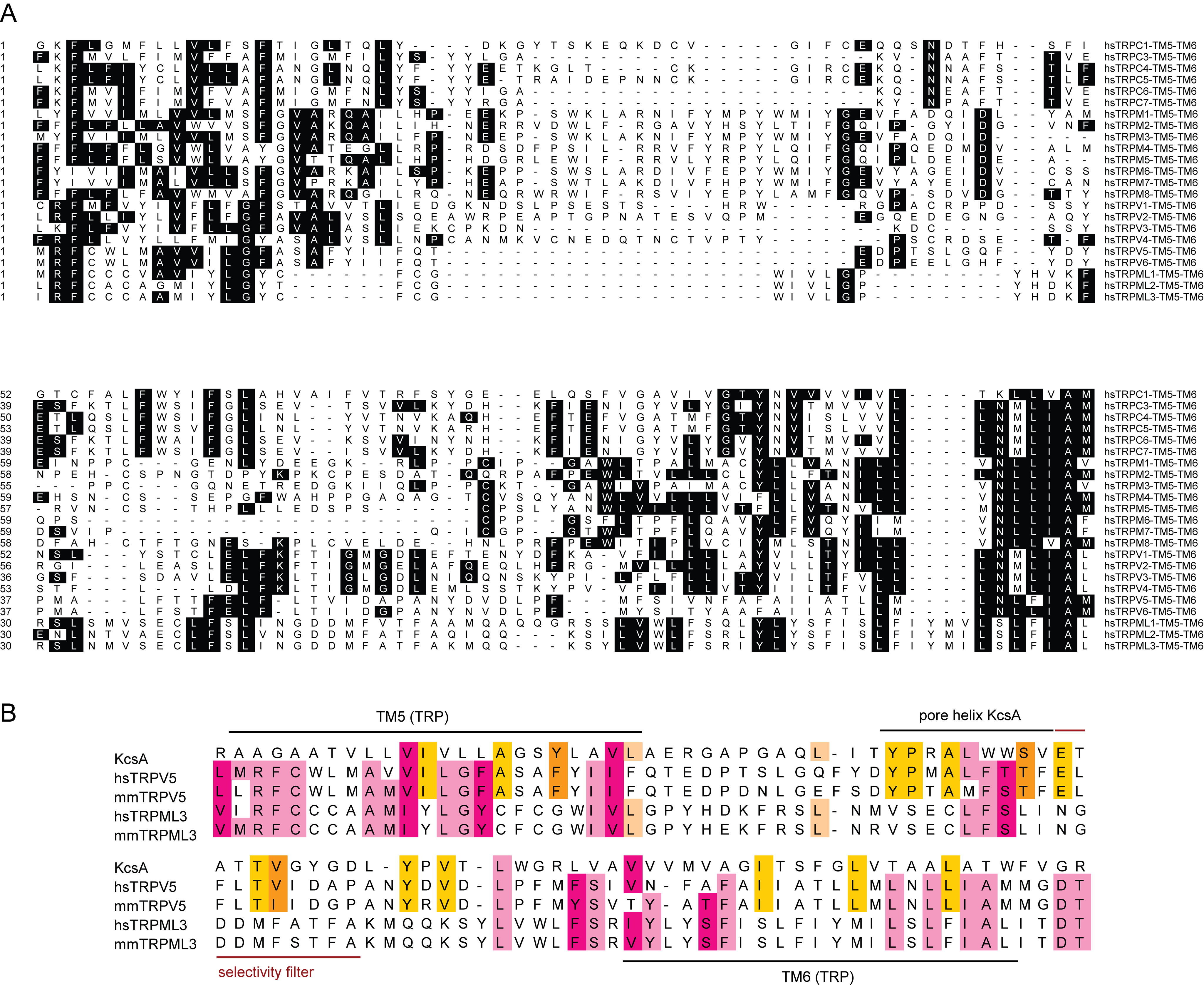

Supplement: Figure S1 — Alignments of TRP channel sequences spanning TM5, the pore loop, and TM6. (A) Identical amino acids are shown with black background. (B) TM5-pore-TM6 sequences of human and mouse TRPV5 and TRPML3. The corresponding sequence of KcsA is shown for comparison. (TIF) [file pone.0058174.s001.tif]

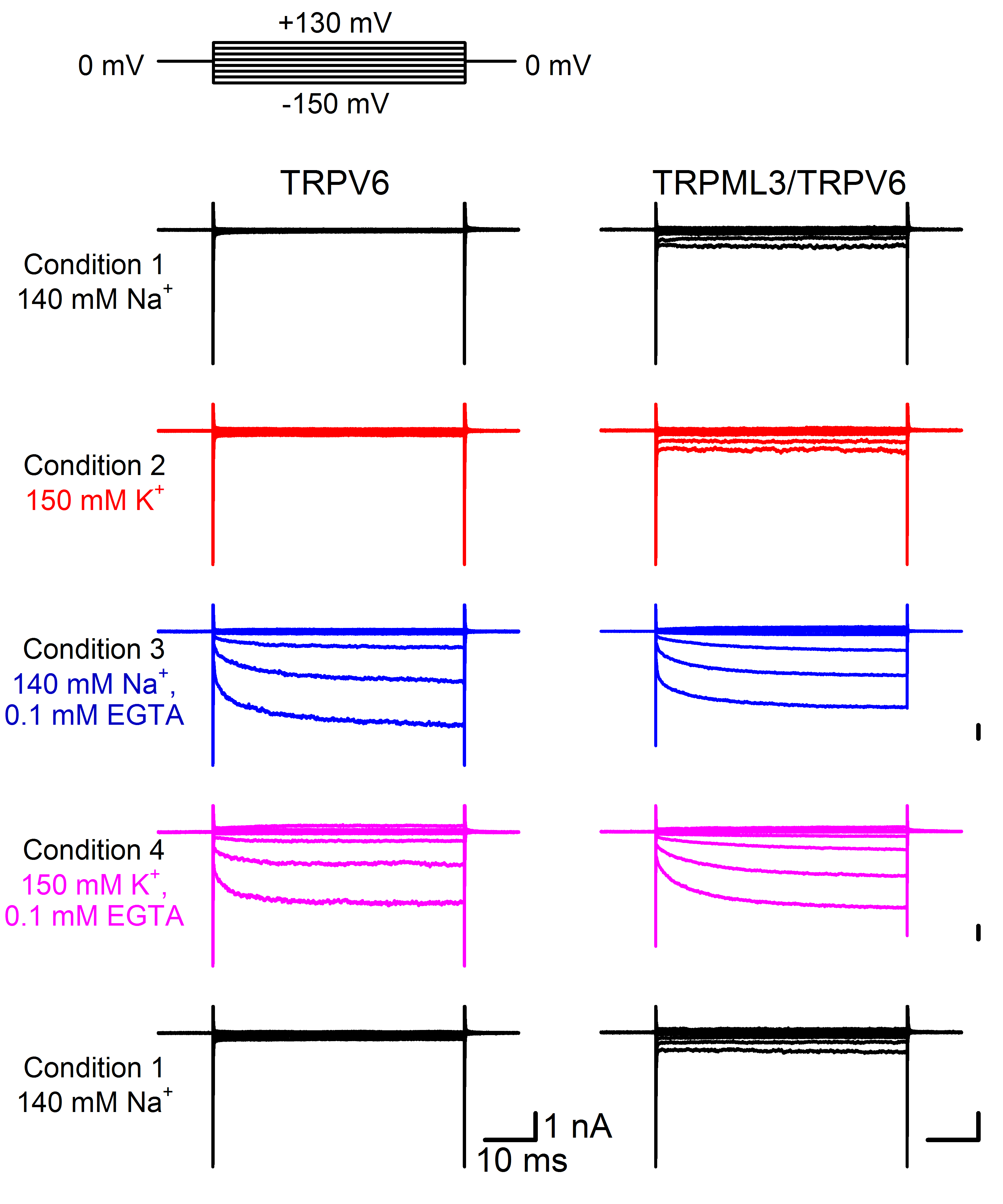

Supplement: Figure S2 — Whole-cell currents of cells expressing TRPV6, and TRPML3 and TRPV6 in different ionic conditions. Traces show representative currents obtained from transfected HEK293 cells expressing TRPV6, and TRPML3 and TRPV6 in the presence of extracellular solutions containing 140 mM Na+ (condition 1, black), 150 mM K+ (condition 2, red), 140 mM Na+, 0.1 mM EGTA (condition 3, blue), and 150 mM K+, 0.1 mM EGTA (condition 4, pink). Currents were recorded during voltage steps from −150 mV to +130 mV in 20 mV increments, holding at 0mV. (TIF) [file pone.0058174.s002.tif]
