# Supplementary material for: A Novel Ion Channel Formed by Interaction of TRPML3 with TRPV5
Source: PLoS One. 2013 Feb 28;8(2):e58174. doi: 10.1371/journal.pone.0058174 (PMC3585263; doi:10.1371/journal.pone.0058174)
Supplement: Table S1 — Accession numbers and sequences of the human TRP protein TM5-pore domain-TM6 segments used in the multiple sequence alignments. (PDF) [file pone.0058174.s003.pdf]

Name	Accession No.	TMD5 to TMD6 regions
TRPC1	NM_003304	GKFLGMFLLVLFSFTIGLTQLYDKGYTSK EQKDCVGIFCEQQSNDTFHSFIGTCFAL FWYIFSLAHVAIFVTRFSYGEELQSFVGA VIVGTYNVVVVIVLTKLLVAMLH
TRPC3	NM_003305	FKFMVLFIMVFFAFMIGMFILYSYYLGAK VNAAFTTVEESFKTLFWSIFGLSEVTSVV LKYDHKFIENIGYVLYGIYNVTMVVVLLN MLIAMIN
TRPC4	NM_016179	LKFLFIYCLVLLAFANGLNQLYFYYEETK GLTCKGIRCEKQNNAFSTLFETLQSLFW SIFGLINLYVTNVKAQHEFTEFVGATMFG TYNVISLVVLLNMLIAMMN
TRPC5	NM_012471	LKFLFIYCLVLLAFANGLNQLYFYYETRAI DEPNNCKGIRCEKQNNAFSTLFETLQSL FWSVFGLLNLYVTNVKARHEFTEFVGAT MFGTYNVISLVVLLNMLIAMMN
TRPC6	NM_004621	FKFMVIFIMVFVAFMIGMFNLYSYYIGAK QNEAFTTVEESFKTLFWAIFGLSEVKSV VINYNHKFIENIGYVLYGVYNVTMVIVLLN MLIAMIN
TRPC7	NM_020389	FKFMVIFIMVFVAFMIGMFNLYSYYRGAK YNPAFTTVEESFKTLFWSIFGLSEVISVV LKYDHKFIENIGYVLYGVYNVTMVVVLLN MLIAMIN
TRPV1	NM_080706	CRFMFVYIVFLFGFSTAVVTLIEDGKNDS LPSESTSHRWRGPACRPPDSSYNSLYS TCLELFKFTIGMGDLEFTENYDFKAVFIIL LLAYVILTYILLLNMLIALMG
TRPV2	NM_016113	LRFLLIYLVFLFGFAVALVSLSQEAWRPE APTGPNATESVQPMEGQEDEGNGAQY RGILEASLELFKFTIGMGELAFQEQLHFR GMVLLLLLAYVLLTYILLLNMLIALMG
TRPV3	NM_145068	LKFLFVYIVFLLGFGVALASLIEKCPKDNK DCSSYGSFSDAVLELFKLTIGLGDLNIQQ NSKYPILFLFLLITYVILTFVLLLNMLIALMS
TRPV4	NM_021625	FRFLLVYLLFMIGYASALVSLLNPCANMK VCNEDQTNCTVPTYPSCRDSETFSTFLL DLFKLTIGMGDLEMLSSTKYPVVFIILLVT YIILTFVLLLNMLIALMG

TRPV5	NM_019841	MRFCWLMAVVILGFASAFYIIFQTEDPTSLGQFYDYPMALFTTFELFLTVIDAPANYDVDLPFMFSIVNFAFAIIATLLMLNLFIAMMG
TRPV6	NM_014274	MRFCWLMAVVILGFASAFYIIFQTEDPEELGHFYDYPMALFSTFELFLTIIDGPANYNVDLPFMYSITYAAFAIIATLLMLNLLIAMMG
TRPM1	NM_002420	LYFVVIMLVVLMSFGVARQAILHPEEKPSWKLARNIFYMPYWMIYGEVFADQIDLYAMEINPPCGENLYDEEGKRLPPCIPGAWLTPALMACYLLVANILLVNLLIAVFN
TRPM2	NM_003307	FFFLFLLAVWVVSFGVAKQAILIHNERRVDWLFRGAVYHSYLTIFGQIPGYIDGVNFNPEHCSPNGTDPYKPKCPESDATQQRPAFPEWLTVLLLCLYLLFTNILLLNLLIAMFN
TRPM3	NM-020952	MYFVIIMLVVLMSFGVARQAILFPNEEPSWKLAKNIFYMPYWMIYGEVFADQIDPPCGQNETREDGKIIQLPPCKTGAWIVPAIMACYLLVANILLVNLLIAVFN
TRPM4	NM_017636	FFFLFFLGVWLVAYGVATEGLLRPRDSDFPSILRRVFYRPYLQIFGQIPQEDMDVALMEHSNCSSEPGFWAHPPGAQAGTCVSQYANWLVVLLLVIFLLVANILLVNLLIAMFS
TRPM5	NM_014555	FFFLFFLSVWLVAYGVTTQALLHPHDGRLEWIFRRVLYRPYLQIFGQIPLDEIDEARVNCSTHPLLLEDSPSCPSLYANWLVILLLVTFLLVTNVLLMNLLIAMFS
TRPM6	NM_017662	FYIVIIMAIVLLSFGVARKAILSPKEPPSWSLARDIVFEPYWMIYGEVYAGEIDVCSSQPSCPPGSFLTPFLQAVYLFVQYIIMVNLLIAFFN
TRPM7	NM_017672	FYIVVIMALVLLSFGVPRKAILYPHEAPSWTLAKDIVFHPYWMIFGEVYAYEIDVCANDSVIPQICGPGTWLTPFLQAVYLFVQYIIMVNLLIAFFN
TRPM8	NM_024080	FFFLFLFAVWMVAFGVARQGILRQNEQRWRWIFRSVIYEPYLAMFGQVPSDVDGTTYDFAHCTFTGNESKPLCVELDEHNLPRFPEWITIPLVCIYMLSTNILLVNLLVAMFG

TRPP1	NM_000297	FGFAIMFFIIFLAYAQLAYLVFGTQVDDFSTFQECIFTQFRIILGDINFAEIEEANRVLGPIYFTTFVFFMFFILLNMFLAIIN
TRPP2	NM_016112	LGFAVMFFIVFFAYAQLGYLLFGTQVENFSTFIKCIFTQFRIILGDFDYNAIDNANRILGPAYFVTYVFFVFFVLLNMFLAIIN
TRPP3	NM_014386	VGFAIMFFIIFFAYAQLGFLVFGSQVDDFSTFQNSIFAQFRIVLGDFNFAGIQQANPILGPIYFITFIFFVFFVLLNMFLAIIN
TRPML1	NM_020533	MRFCCCVAVIYLGYCFCGWIVLGPYHVKFRSLSMVSECLFSLINGDDMFVTFAAMQAQQGRSSLVWLFSQLYLYSFISLFIYMVLSLFIALIL
TRPML2	XM_351263	LRFCACAGMIYLGYTFCGWIVLGPYHDKFENLNTVAECLFSLVNGDDMFATFAQIQQKSILVWLFSRLYLYSFISLFIYMILSLFIALIL
TRPML3	NM_018298	IRFCCCAAMIYLGYCFCGWIVLGPYHDKFRSLNMVSECLFSLINGDDMFATFAKMQQKSYLVWLFSRIYLYSFISLFIYMILSLFIALIL
